# Social exclusion in educational settings and the risk of alienation in adolescent development: a narrative review from a mind-body interaction perspective

**DOI:** 10.3389/fpsyg.2026.1807335

**Published:** 2026-10-01

**Authors:** Di Lu, Yumeng Kong, Jinwu Tang

**Affiliations:** 1 College of Marxism, Qufu Normal University, Jining, China; 2 College of Sports Science, Qufu Normal University, Jining, China

**Keywords:** adolescence, social exclusion, educational settings, alienation, mind-body interaction, peripheral inflammation, neuroimmune signaling, neural plasticity

## Abstract

**Introduction:**

Social exclusion in educational settings is a salient interpersonal stressor during adolescence. However, ostracism, peer rejection, peer victimization, bullying, loneliness, and social isolation are overlapping but non-equivalent constructs. This narrative review examines how social exclusion in educational settings may contribute to the risk of alienation in adolescent development from a mind-body interaction perspective.

**Methods:**

A structured, non-exhaustive literature search was conducted in PubMed, PsycINFO, and the Web of Science Core Collection for peer-reviewed English-language publications from 1 January 2000 to 21 July 2026, supplemented by backward citation tracking. Evidence was organized by construct, population, study design, and biological level. Direct adolescent human findings were distinguished from related adolescent, adult or clinical human, and animal or cellular evidence.

**Results:**

Direct adolescent studies indicate that peer victimization and interpersonal stress can be associated with heightened peripheral cytokine reactivity, particularly among youths with additional cognitive or developmental vulnerabilities. Developmental neuroscience and neuroimmunology identify plausible endothelial, vagal, glial, synaptic, white-matter, hippocampal, and network pathways through which immune signals could interact with adolescent brain maturation. Nevertheless, the literature does not establish a continuous causal sequence from school exclusion to central neuroinflammation, altered neural plasticity, alienation, or educational impairment.

**Discussion:**

The evidence supports a conditional, evidence-graded mind-body framework rather than a deterministic pathway. Educational practice should prioritize reducing exclusion, strengthening belonging and social support, and providing accessible psychological support; general health-promoting strategies should remain complementary. Longitudinal, multimodal, and developmentally stratified studies are needed to test temporal ordering, mediation, and resilience.

## 1 Introduction

Social exclusion is a broad social and interpersonal phenomenon with substantial implications for adolescent mental health, wellbeing, and participation in education (Filia et al., 2025; Williams and Nida, 2022). At the population level, exclusion can occur across relational, educational and employment, financial, housing, and service-access domains (Filia et al., 2025). In a national survey of 18,800 Australian young people aged 15–19 years, 60% reported exclusion in at least one domain and 25% reported exclusion in multiple domains; difficulties in socialising and obtaining social support were particularly associated with greater psychological distress and reduced wellbeing (Filia et al., 2025). These figures establish the public-health importance of social exclusion, but they should not be interpreted as indicating the prevalence of peer ostracism alone because the survey adopted a multidomain social-determinants framework (Filia et al., 2025). The present review therefore narrows its primary focus to interpersonal exclusion in educational settings, including being ignored, left out, rejected, or excluded from peer interaction, while treating bullying, loneliness, and objective social isolation as related but distinct constructs.

For this review, adolescence is operationally defined as 10–19 years, consistent with the definition adopted by World Health Organization (2024). Studies extending beyond this age range are identified explicitly as young-adult or lifespan evidence. This distinction matters because adolescence is not a homogeneous developmental stage. Pubertal timing, endocrine changes, social reorientation, and the ongoing maturation of prefrontal, limbic, hippocampal, and white-matter systems vary across early, middle, and late adolescence (Holder and Blaustein, 2014; Larsen and Luna, 2018; Baker et al., 2025). Recent lifespan imaging work further indicates that structural brain topology changes non-linearly rather than following a uniform maturational trajectory, underscoring the need to avoid treating adolescent neurodevelopment as a single linear process (Mousley et al., 2025). Experimental evidence in mice also indicates that prefrontal microglia contribute to cognitive and synaptic maturation during adolescence (Schalbetter et al., 2022). However, these animal findings cannot be assumed to demonstrate an equivalent mechanism in human adolescents.

Social exclusion is especially relevant during adolescence because peer evaluation and social belonging acquire heightened motivational and emotional salience during this developmental period (Somerville, 2013). Experimental exclusion tasks indicate that adolescents differ substantially in their neural and emotional responses to exclusion (Masten et al., 2009). Longitudinal evidence further suggests that a history of chronic peer rejection during childhood is associated with heightened neural responses to exclusion during adolescence (Will et al., 2016b). Rejection sensitivity represents an additional individual-difference factor associated with adolescent emotional-disorder symptoms and maladaptive responses to anticipated rejection (Minihan et al., 2023). Cognitive vulnerability may also intensify adolescents’ inflammatory responses following peer victimization and acute social stress (Giletta et al., 2018). These findings support the view that social exclusion is not merely an unpleasant event but a context-dependent social-evaluative stressor whose effects vary across individuals and developmental stages.

A useful theoretical starting point is the Social Signal Transduction Theory of Depression, which proposes that experiences of social threat are represented in neural systems involved in threat, salience, and distress processing and are subsequently translated into autonomic, endocrine, and immune changes (Slavich and Irwin, 2014). Related social-neuroscience models likewise describe how threats to social connection can engage neural and physiological alarm systems with downstream implications for physical and mental health (Eisenberger and Cole, 2012). These frameworks support the plausibility of a pathway from social-evaluative threat to peripheral inflammatory activity, but they do not by themselves establish that exclusion within educational settings causes central neuroinflammation or lasting neural damage.

The empirical literature is fragmented across levels of analysis. Some adolescent studies assess peer victimization together with salivary inflammatory responses to laboratory social stress (Giletta et al., 2018). Other adolescent studies assess early-life adversity together with circulating inflammatory markers and stress-related immune gene expression (Kuhlman et al., 2022). Longitudinal adolescent research has also examined interactions between interpersonal life stress, inflammatory reactivity, and subsequent depressive symptoms (Slavich et al., 2020). In contrast, other human studies examine loneliness and objective social isolation in adult or lifespan cohorts rather than school-based peer exclusion during adolescence (Matthews et al., 2024). Animal and translational studies provide additional evidence concerning social-stress-induced immune sensitization, but their findings cannot be treated as direct evidence concerning school exclusion in human adolescents (Biltz et al., 2022). Separate developmental-neuroscience and neuroimmunology literatures address microglial contributions to adolescent prefrontal development (Schalbetter et al., 2022). Other work describes the context-dependent roles of cytokines in learning, memory, and synaptic plasticity (Bourgognon and Cavanagh, 2020). Developmental imaging research further examines how adolescent brain connectivity supports learning and experience-dependent development (Baker et al., 2025). Meta-analytic evidence also describes the contribution of amygdala–prefrontal connectivity to emotion regulation, although such evidence is not specific to social exclusion or inflammation in adolescents (Berboth and Morawetz, 2021). The central methodological challenge is therefore not simply to assemble these findings into a linear narrative, but to determine which links are directly supported in adolescents, which are extrapolated from other populations or experimental models, and where evidence remains absent.

Accordingly, this narrative review has four aims. First, it defines social exclusion and distinguishes it from related constructs used in the literature. Second, it evaluates evidence linking adolescent social stress with peripheral immune responses and separates peripheral biomarkers from evidence of central neuroimmune alterations. Third, it examines whether developmental neuroscience provides plausible—but not necessarily direct—mechanisms through which sustained immune signaling could influence adolescent neural plasticity. Fourth, it considers educational implications while distinguishing interventions that reduce exclusion from general health-promoting strategies. Throughout the review, causal language is reserved for experimental evidence, and each inferential step is labelled according to the population, model, and study design supporting it.

## 2 Review approach

This article was conducted as a narrative review supported by a structured, non-exhaustive literature search. It was not designed as a systematic review or meta-analysis, and no review protocol was registered. The purpose of the search was to identify and critically integrate representative evidence across social psychology, developmental neuroscience, psychoneuroimmunology, education, and neuroimmunology. Particular attention was given to the strength and limitations of each inferential link in the proposed pathway, rather than to presenting the pathway as an established causal sequence.

Literature searches were conducted in PubMed, PsycINFO, and the Web of Science Core Collection for peer-reviewed English-language publications issued between 1 January 2000 and 21 July 2026. Search syntax was adapted to the indexing requirements of each database. Three complementary search streams were used because a single search requiring simultaneous coverage of social exclusion, inflammation, and neural plasticity would have excluded studies examining only one component of the proposed pathway. The first search stream targeted adolescent social exclusion and peripheral immune activity using the following combination: (“social exclusion” OR ostracism OR “peer rejection” OR “peer victimization” OR “relational bullying” OR cyberexclusion) AND (adolescen* OR youth OR teen*) AND (inflamm* OR cytokine* OR immun* OR “C-reactive protein” OR CRP OR IL-6 OR IL-1β OR TNF-α). The second search stream targeted inflammation and adolescent neural plasticity: (adolescen* OR youth OR puberty) AND (inflamm* OR cytokine* OR microglia OR neuroimmune) AND (“synaptic plasticity” OR LTP OR LTD OR neurogenesis OR myelination OR “white matter” OR prefrontal OR hippocamp* OR amygdala). The third search stream targeted social exclusion, brain function, and educational outcomes: (“social exclusion” OR ostracism OR “peer rejection” OR “peer victimization” OR “relational bullying” OR cyberexclusion) AND (adolescen* OR youth OR teen*) AND (brain OR fMRI OR neuroimaging OR cognition OR “executive function” OR academic OR school OR “educational outcome”). Additional targeted searches addressed school belonging, social-evaluative threat, vagal immune signaling, brain endothelial signaling, blood–brain barrier regulation, cytokine-dependent synaptic plasticity, neurotransmitter systems, and adolescent microglial development. The reference lists of relevant original studies, systematic reviews, meta-analyses, and major theoretical articles were also screened to identify additional publications.

Priority was given to original human studies involving participants aged 10–19 years. Studies were considered direct adolescent evidence when they operationalized interpersonal social exclusion, ostracism, peer rejection, peer victimization, exclusionary or relational bullying, or cyberexclusion and assessed at least one relevant peripheral immune, neuroimaging, cognitive, psychological, social, or educational outcome. Studies examining broader interpersonal stress or early-life adversity in adolescents were retained as related adolescent evidence but were not treated as direct evidence of school-based social exclusion. Adult human studies, clinical samples, lifespan studies, and animal or cellular models were included only when they clarified a mechanistic link that could not currently be evaluated directly in human adolescents. These sources were explicitly identified as indirect human, clinical, animal, or cellular evidence. Review articles were used to establish theoretical context and identify original studies, but the principal empirical claims were supported preferentially by original research. Studies of infection, autoimmune disease, experimentally induced physical inflammation, adulthood, or aging were not treated as direct evidence that social exclusion alters adolescent neurodevelopment.

For each study used to support a major inferential link, the exposure construct, species, participant age range, sample size, study design, biological specimen, inflammatory marker, central nervous system measure, principal finding, and major limitation were extracted. Studies were classified into four evidence categories: direct adolescent human evidence, related adolescent human evidence, indirect adult or clinical human evidence, and animal or cellular evidence. The synthesis was organized according to these evidence levels rather than by assuming that the complete sequence from social exclusion to inflammation, altered neural plasticity, and psychological or educational outcomes had been demonstrated. The database searches identified 2,433 records. After deduplication and staged title/abstract and full-text relevance screening, 105 sources were retained in the final narrative synthesis. Four seminal studies published before 2000 were additionally identified through backward citation tracking because they provided foundational construct definitions or original mechanistic evidence that had not been superseded by later publications. These studies were used only for foundational or mechanistic context and did not alter the primary 2000–2026 database-search window. Because this was a narrative review with iterative and mechanism-oriented searches, the counts are reported to improve transparency, but a PRISMA flow diagram and formal meta-analytic risk-of-bias assessment were not used. The original studies supporting the central inferential links are summarized in the evidence table, with their populations, measurements, findings, and limitations reported explicitly. The evidence classification and study characteristics were independently checked by two authors, and disagreements were resolved through discussion.

## 3 Conceptual scope and theoretical framework

### 3.1 Defining social exclusion and related constructs

For the purposes of this review, social exclusion is used as an umbrella term for experiences in which an adolescent is ignored, omitted, or prevented from participating in desired social interaction. Ostracism refers more specifically to being ignored or excluded by other individuals or groups, and it may occur during either brief interactions or sustained interpersonal relationships (Williams and Nida, 2022).

Peer rejection refers to a sociometric or group-level status characterized by relatively low acceptance and high dislike among peers (Coie et al., 1982). Measures of peer acceptance, rejection, and social preference show substantial short-term stability and meaningful longer-term stability across childhood and adolescence, although their stability varies according to age, sex, measurement interval, and study characteristics (Jiang and Cillessen, 2005). Peer victimization refers to experiences in which a child or adolescent is the target of aggressive acts committed by peers, including physical, verbal, or relational aggression (Perry et al., 1988). Peer victimization is broader than bullying because not every aggressive peer interaction necessarily involves repetition or an imbalance of power. Bullying should not be treated as synonymous with either social exclusion or general peer victimization. Bullying is conventionally defined by three central characteristics: an intention to cause harm, repetition over time, and an imbalance of power that makes it difficult for the targeted individual to defend himself or herself (Menesini and Salmivalli, 2017). Within the present review, bullying evidence is considered directly relevant only when the behavior includes relational or exclusionary practices, such as deliberately removing an adolescent from a friendship group, spreading information intended to damage relationships, or preventing participation in group activities (Casper et al., 2020).

Social isolation and loneliness also require separate definitions. Social isolation generally denotes an objective lack or scarcity of social contacts and interactions with family members, friends, or the wider community, whereas loneliness denotes a subjective negative experience arising from a perceived discrepancy between desired and actual social relationships (Valtorta and Hanratty, 2012). Although this distinction has often been discussed in adult health research, it is conceptually applicable to adolescent studies: an adolescent may have few social contacts without feeling lonely or may experience loneliness despite participating in an apparently extensive social network. Cyberostracism or cyberexclusion refers to actual or perceived experiences of being ignored or excluded through internet-based or other digitally mediated forms of communication (Williams et al., 2000). It can include being ignored in online conversations, excluded from digital groups, receiving no response after a message has been viewed, or receiving substantially less social feedback than other group members (Schneider et al., 2017).

These constructs are conceptually related but should not be treated as interchangeable. A laboratory ostracism task typically models an acute and experimentally controlled exclusion episode (Williams and Nida, 2022). By contrast, peer rejection can represent a comparatively stable position within an established peer group (Jiang and Cillessen, 2005). Exclusionary bullying may involve intentional and repeated behavior embedded in an ongoing imbalance of power within the school environment (Menesini and Salmivalli, 2017). Loneliness may develop following exclusion but can also occur in the absence of objective isolation, while objectively isolated individuals do not necessarily report loneliness (Valtorta and Hanratty, 2012). Each study discussed below is therefore classified according to its operational construct, and its findings are not generalized to other forms of social disconnection without explicit qualification. Table 1 outlines operational distinctions among social exclusion and related constructs.

**Table 1:** Operational distinctions among social exclusion and related constructs.

Construct	Operational definition	Distinguishing features	Typical measurement or operationalization	Use in the present review and supporting reference(s)
Social exclusion	A working umbrella term for experiences in which an adolescent is ignored, omitted, or prevented from participating in desired social interaction.	This is the broad organizing construct used in the review. It does not imply that ostracism, peer rejection, bullying, loneliness, and objective isolation are equivalent.	Self-report, peer report, teacher report, observation, sociometric indicators, or experimental exclusion tasks; the specific operationalization must be reported.	Primary umbrella construct. Findings are interpreted according to the more specific exposure actually measured (Williams and Nida, 2022)
Ostracism	Being ignored or excluded by other individuals or groups. It may occur in a brief interaction or across a longer interpersonal relationship.	Ostracism emphasizes being ignored or excluded. Experimental paradigms usually model an acute episode and should not automatically be treated as equivalent to chronic school exclusion.	Cyberball, Ostracism Online, recall or vignette paradigms, daily-diary measures, and self-reported experiences of being ignored or left out.	Direct exclusion construct, but the duration and ecological context must be specified (Williams and Nida, 2022)
Peer rejection	A sociometric or group-level status characterized by relatively low acceptance and high dislike among peers.	Peer rejection describes a position within a peer group rather than a single exclusion event. It may be comparatively stable, although stability varies by age, sex, measurement interval, and study characteristics.	Peer nominations, ratings of liked-most and liked-least classmates, social preference scores, and peer-acceptance or rejection indices.	Direct, relatively chronic group-level construct (Coie et al., 1982; Jiang and Cillessen, 2005)
Peer victimization	Being the target of aggressive acts committed by peers, including physical, verbal, or relational aggression.	Peer victimization is broader than bullying because an aggressive act does not always meet the criteria of repetition and power imbalance. It is also broader than exclusion because the aggressive act may not involve social omission or rejection.	Self-report, peer nomination, teacher report, interview, school incident records, and victimization questionnaires that specify overt, verbal, or relational forms.	Related construct. Evidence is treated as directly relevant only when the victimization includes exclusionary or relational content (Perry et al., 1988)
Exclusionary or relational bullying	Intentional and repeated relational aggression occurring within a power imbalance and involving exclusion, removal from a group, or damage to social relationships.	Bullying is not synonymous with social exclusion. Only bullying that includes relational or exclusionary practices is treated as construct-matched evidence in this review.	Bullying questionnaires, peer or teacher reports, school records, and relational-aggression measures that assess repetition, intentionality, power imbalance, and exclusionary content.	Directly relevant only when exclusionary behavior is explicitly measured (Menesini and Salmivalli, 2017; Casper et al., 2020)
Social isolation	An objective scarcity of social contacts, interactions, or network connections.	Social isolation is an objective social condition and does not necessarily imply loneliness. It may arise without an identifiable exclusion event.	Social-network size, contact frequency, number of close relationships, participation in social activities, and structural network indices.	Indirect construct unless the study links isolation to prior exclusion or rejection. The cited source is adult-focused and is used for conceptual distinction rather than adolescent-specific empirical inference (Valtorta and Hanratty, 2012)
Loneliness	A subjective negative experience arising from a perceived discrepancy between desired and actual social relationships.	Loneliness can follow exclusion but can also occur despite an objectively large social network. It should not be used as a synonym for objective isolation or peer rejection.	Validated self-report measures of perceived loneliness or unmet relationship needs, such as the UCLA Loneliness Scale or age-adapted loneliness scales.	Potential consequence, correlate, or moderator rather than a direct substitute for exclusion. The cited source is adult-focused and is used for the conceptual distinction (Valtorta and Hanratty, 2012)
Cyberexclusion or cyberostracism	Actual or perceived experiences of being ignored or excluded through internet-based or digitally mediated communication.	This is a digital form of exclusion. It should be distinguished from broader cyberbullying, which may involve harassment, threats, or reputational aggression rather than exclusion.	Online experimental paradigms, social-media ostracism tasks, self-report of being omitted from digital groups, unanswered messages, or receiving markedly less feedback than peers.	Direct online form of exclusion; the platform, behavior, duration, and comparison condition should be reported (Williams et al., 2000; Schneider et al., 2017)

### 3.2 Evidence hierarchy and inferential boundaries

The proposed pathway is evaluated using an evidence hierarchy. Level 1 evidence comprises longitudinal or experimental adolescent human studies that directly assess the relevant exposure and biological outcome. Level 2 evidence comprises adolescent human studies that assess a related social stressor, peripheral biomarker, or neural outcome but do not test the full link. Level 3 evidence comprises adult or clinical human evidence. Level 4 evidence comprises animal or cellular mechanism studies. Arrows in the conceptual model are labeled with these levels so that a plausible mechanism is not visually presented as a demonstrated causal sequence.

### 3.3 Social signal transduction framework

Within the evidence hierarchy described above, the Social Signal Transduction Theory of Depression provides the principal psychological-to-biological framework for the present review (Slavich and Irwin, 2014). The theory proposes that experiences involving social threat and adversity are represented in neural systems involved in threat detection, salience, and distress and can subsequently influence sympathetic nervous system activity, hypothalamic–pituitary–adrenal axis activity, proinflammatory gene expression, and peripheral inflammatory signaling (Slavich and Irwin, 2014). Related social-neuroscience models similarly propose that threats to social connection can engage neural and physiological alarm systems that translate experiences of social disconnection into health-relevant biological responses (Eisenberger and Cole, 2012).

This framework is relevant to the present review because social evaluation, rejection, isolation, and exclusion are forms of interpersonal threat explicitly discussed within Social Signal Transduction Theory (Slavich and Sacher, 2019). However, the framework should not be interpreted as a deterministic sequence in which every experience of social exclusion necessarily produces persistent inflammation, central neuroimmune alterations, or neural injury. A meta-analysis of childhood and adolescent stress studies found a small overall association between early-life psychological stress and inflammatory activity, together with substantial heterogeneity across studies and developmental stages (Chiang et al., 2022).

The magnitude of stress-related biological responses may depend partly on how the event is appraised and on the adolescent’s prior exposure to adversity (Slavich and Irwin, 2014). Adolescent evidence indicates that early-life adversity may be associated with altered inflammatory and immune gene-expression responses to acute social stress, although early adversity should not be treated as equivalent to school-based social exclusion (Kuhlman et al., 2022). Social support may buffer stress-system activity, but the source and effectiveness of such support change across development (Gunnar and Hostinar, 2015).

Sex-related biological processes and pubertal hormonal changes may also influence stress sensitivity and inflammatory activity, although these effects are complex and should not be presented as uniform sex differences (Slavich and Sacher, 2019). Sleep disturbance may be associated with increased inflammatory activity and may interact with psychosocial stress, but the available evidence concerning sex differences remains inconsistent (Dolsen et al., 2019). Adiposity must also be considered when interpreting adolescent inflammatory markers because body-fat percentage is associated with markers including C-reactive protein, leptin, and adiponectin (Rocha et al., 2025). Accordingly, the proposed pathway should be understood as conditional, developmentally sensitive, and potentially recursive rather than as a single inevitable progression from exclusion to inflammation and neural impairment. Figure 1 shows Conceptual scope and evidence hierarchy for the proposed social exclusion–immune signaling pathway.

**Figure 1:**
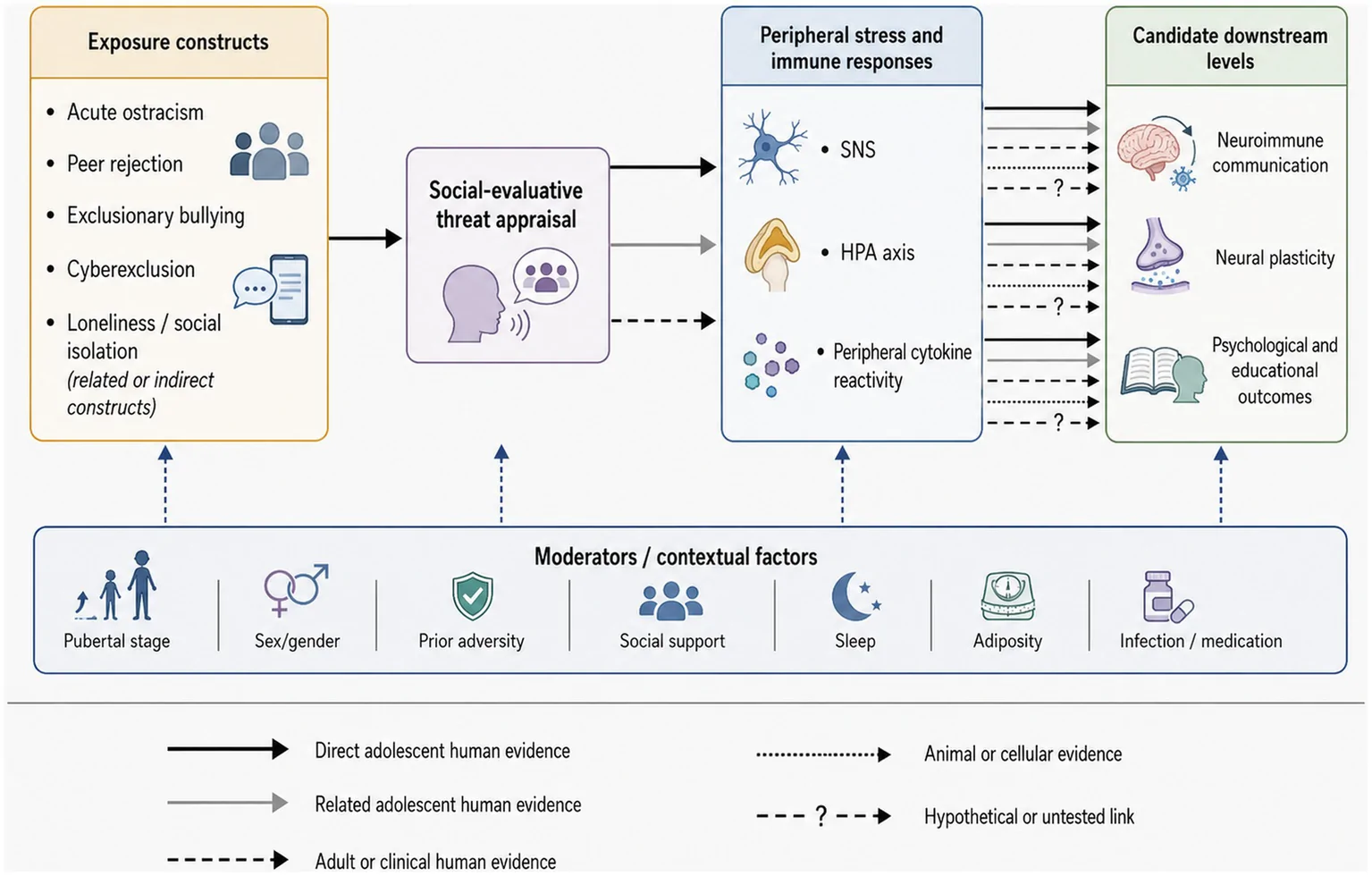
Conceptual scope and evidence hierarchy for the proposed social exclusion–immune signaling pathway.

The figure distinguishes acute ostracism, peer rejection, exclusionary bullying, cyberexclusion, loneliness, and objective social isolation. Arrows are coded by evidence level: solid dark arrows indicate direct adolescent human evidence; solid light arrows indicate related adolescent human evidence; dashed arrows indicate adult or clinical human evidence; and dotted arrows indicate animal or cellular evidence. The figure represents an inferential framework and does not imply that the complete pathway has been established causally. Source: Created by the authors using original schematic artwork; no third-party copyrighted material is reproduced.

## 4 Human evidence linking adolescent social stress to peripheral immune activity

First, direct adolescent human evidence links peer victimization with peripheral inflammatory reactivity under acute social stress. Giletta and colleagues studied 157 adolescent girls at elevated risk for psychopathology, with a mean age of 14.73 years, using a laboratory social-stress task. Greater prior peer victimization predicted larger stress-related increases in salivary IL-6 and IL-1β, particularly among adolescents reporting greater hopelessness (Giletta et al., 2018). This result gives empirical support to a subgroup-sensitive social-stress model in which cognitive appraisal helps shape peripheral immune reactivity. Because the study used salivary markers and a selected female sample, it most directly informs the acute peripheral component of the framework.

Relatedly, Kuhlman and colleagues examined a community sample of adolescents with a mean age of 13.9 years using the Trier Social Stress Test for Children. Blood samples collected before and after the task showed larger stress-associated increases in proinflammatory and innate antiviral gene transcripts among adolescents with higher exposure to early-life adversity; the same group showed a smaller initial but more prolonged increase in circulating IL-6 (Kuhlman et al., 2022). These findings extend the developmental stress-sensitization literature by showing that adversity-related differences can be expressed in both inflammatory gene transcription and circulating cytokine dynamics. In the present evidence hierarchy, they inform a related adolescent-stress link, while early-life adversity is kept distinct from school-based social exclusion.

Complementing these findings, a nine-month longitudinal study examined 116 adolescent girls at elevated risk for psychopathology. Greater interpersonal life-stress exposure predicted increases in depressive symptoms among girls with stronger salivary TNF-α or IL-1β reactivity to a laboratory stressor, but not among girls with weaker reactivity; the interaction was not observed for IL-6 (Slavich et al., 2020). The study therefore supports inflammatory reactivity as a possible moderator of interpersonal-stress vulnerability and highlights the value of testing conditional, rather than uniform, biological responses.

By contrast, Matthews and colleagues provide contextual lifespan evidence rather than direct evidence on school-based adolescent exclusion. In the adult TRIAGE cohort, living alone was associated with higher soluble urokinase plasminogen activator receptor (suPAR) but not higher CRP or IL-6; in the E-Risk cohort assessed at age 18, loneliness was not associated with elevated inflammatory markers (Matthews et al., 2024). This contrast is informative because it shows that social-connection constructs and inflammatory markers do not have uniform relationships across developmental and clinical contexts. The study therefore helps define the boundary of adolescent inference: objective isolation and loneliness are related to, but not interchangeable with, peer exclusion.

Taken together, direct adolescent evidence supports a conditional association between peer victimization, interpersonal stress, developmental adversity, and peripheral inflammatory reactivity in some adolescents, particularly those with additional cognitive or developmental vulnerabilities (Giletta et al., 2018; Kuhlman et al., 2022; Slavich et al., 2020). The convergence is strongest at the level of peripheral reactivity and subgroup differences. It does not yet specify how school-based exposure could connect to central neuroimmune signaling, neural plasticity, or educational outcomes; those downstream links are therefore treated in the remainder of the review as mechanistic hypotheses that organize future testing. Table 2 outlines representative evidence and the inferential link supported by each study.

**Table 2:** Representative evidence and the inferential link supported by each study.

Study	Exposure construct	Population/model	Study design and measures	Main finding	Inferential link supported	Evidence category	Key limitations
Giletta et al. (2018)	Peer victimization	Human adolescents; 157 girls at elevated risk for psychopathology; mean age 14.73 years	Laboratory social stressor; salivary IL-6, IL-1β, and TNF-α assessed before and after the task	Greater prior peer victimization predicted larger stress-related increases in IL-6 and IL-1β. Inflammatory responding was strongest among adolescents with higher hopelessness.	Peer victimization → heightened peripheral cytokine reactivity to acute social stress	Direct/near-direct adolescent human evidence	Female, selected high-risk sample; salivary rather than circulating biomarkers; acute response only; no CNS, microglial, imaging, or plasticity measures
Kuhlman et al. (2022)	Early-life adversity; not school-based exclusion	Community sample of adolescents; mean age 13.9 years; 65 with complete gene-expression data and 84 with complete circulating-marker data	Trier Social Stress Test for Children; blood collected before and 60 and 90 min after task initiation; inflammatory gene expression and circulating markers assessed	High-adversity adolescents showed larger stress-associated increases in proinflammatory and innate antiviral gene transcripts, together with a smaller initial but more prolonged IL-6 response.	Developmental adversity/stress sensitization → altered peripheral immune response to acute social stress	Related adolescent human evidence	Exposure is heterogeneous early-life adversity, not peer exclusion; different analytic subsamples; no CNS measure; circulating-protein findings were not uniformly elevated
Slavich et al. (2020)	Interpersonal life stress	Human adolescents; 116 girls at elevated risk for psychopathology; mean age 14.71 years	Baseline laboratory social stressor with salivary TNF-α, IL-1β, and IL-6 reactivity; interview-assessed life stress and depressive symptoms over 9 months	Greater interpersonal stress predicted increases in depressive symptoms only among girls with stronger TNF-α or IL-1β reactivity. The corresponding interaction was not significant for IL-6.	Inflammatory reactivity moderates the prospective association between interpersonal stress and depressive symptoms	Prospective related adolescent human evidence	Moderation, not mediation; all-female high-risk sample; stressors were broader than school exclusion; no central neuroimmune or neural-plasticity measures
Matthews et al. (2024)	Objective social isolation and loneliness	E-Risk cohort assessed at age 18, with adult and clinical comparator cohorts	Longitudinal and cross-sectional analyses of social isolation or loneliness with CRP, IL-6, and suPAR	Loneliness at age 18 was not associated with elevated inflammatory markers; associations in older or clinical cohorts were strongest for suPAR (Matthews et al., 2024).	Social isolation/loneliness and systemic inflammatory activity as contextual lifespan evidence	Indirect adult/lifespan human evidence	Exposure differs from peer exclusion; adult and clinical comparator data; peripheral markers only; construct and developmental differences limit adolescent inference
Masten et al. (2009)	Experimentally induced social exclusion	Human adolescents; *n* = 23	Cyberball social-exclusion task during fMRI; self-reported exclusion distress and rejection sensitivity; parent-reported interpersonal competence	Exclusion-related distress was positively associated with insular and subgenual anterior cingulate activity, whereas right ventrolateral prefrontal and ventral striatal activity was associated with less distress.	Acute social exclusion → neural and emotional response during adolescence	Direct adolescent human neural evidence	Small sample; brief artificial exclusion task; no inflammatory biomarkers; no longitudinal or educational outcome; neural activation does not establish neuroinflammation
Schalbetter et al. (2022)	Transient prefrontal microglial depletion; not social stress	Adolescent mouse model	Cell-specific and transient depletion of prefrontal microglia during adolescence; later assessment of cognition, dendritic complexity, synaptic structure, and excitation–inhibition balance	Adolescent, but not adult, prefrontal microglial depletion produced lasting impairments in prefrontal-associated cognition, dendritic complexity, synaptic structure, and circuit balance.	Adolescent prefrontal microglia → developmental plasticity and later cognitive function	Animal mechanistic evidence	Experimental depletion does not model exclusion or stress-induced inflammation; species translation is uncertain; no school, peer, or peripheral-inflammatory exposure

“Direct” and “related” refer to construct and population match, not to proof of the complete causal pathway. Peripheral cytokine or inflammatory markers do not by themselves demonstrate central neuroinflammation.

## 5 Peripheral-to-central neuroimmune communication

To interpret the downstream literature precisely, the present review distinguishes among peripheral immune activation, neuroimmune communication, and central neuroinflammation. Peripheral immune activation refers to changes measured in blood, saliva, or peripheral immune cells, including alterations in IL-6, IL-1β, TNF-α, C-reactive protein, or inflammatory gene expression. Neuroimmune communication refers to the neural, humoral, endothelial, and cellular processes through which the peripheral immune system and the central nervous system exchange information. Central neuroinflammation refers more specifically to inflammatory processes occurring within the brain or other central nervous system compartments and requires direct or convergent central evidence, such as brain tissue, cerebrospinal fluid, appropriately interpreted molecular imaging, or central molecular and cellular measures. Peripheral and central inflammatory markers show only limited correspondence across paired blood and cerebrospinal-fluid samples (Gigase et al., 2023). This distinction allows peripheral findings to remain informative without being overextended to the brain.

Mechanistically, peripheral immune signals can influence the brain without requiring unrestricted cytokine passage through a structurally damaged blood–brain barrier. Some circulating cytokines can undergo regulated or saturable transport across blood–brain interfaces, and experimental immune challenge can induce prostaglandin-synthesis enzymes in brain endothelial cells (Banks et al., 1989; Inoue et al., 2002). Neural communication through vagal afferents provides an additional route by which peripheral cytokine activity can be detected and relayed to the brain (Jin et al., 2024). Under more intense, prolonged, or pathological inflammatory conditions, altered barrier permeability and immune-cell trafficking may provide further routes of central immune influence. The available evidence therefore supports several regulated peripheral-to-central communication mechanisms rather than a single leakage model.

In parallel, the vagus nerve should be understood as part of a bidirectional immunoregulatory circuit rather than as a purely proinflammatory conduit. In mice exposed to peripheral immune challenges, distinct populations of vagal sensory neurons responded to proinflammatory and anti-inflammatory cytokines and conveyed this information to the caudal nucleus of the solitary tract; manipulating the circuit altered the magnitude and direction of the peripheral inflammatory response (Jin et al., 2024). These findings identify a homeostatic body–brain circuit that can be evaluated in future developmental studies.

Developmentally, microglia are central nervous system-resident immune cells with substantial spatial, temporal, and disease-related heterogeneity (Masuda et al., 2019). During normal development, they participate in activity-dependent synaptic remodeling, circuit refinement, and trophic support. In mice, microglial engulfment of presynaptic inputs contributes to retinogeniculate connectivity, and microglia-derived IGF-1 supports neuronal survival (Schafer et al., 2012; Ueno et al., 2013). The adolescent period may be especially sensitive: transient, cell-specific depletion of prefrontal microglia during adolescence, but not adulthood, produced later changes in cognition, dendritic complexity, synaptic structure, and excitation–inhibition balance (Schalbetter et al., 2022). This evidence makes developmental microglial timing a plausible bridge between immune signaling and adolescent circuit maturation and motivates the next section’s focus on neural plasticity. The review consequently treats microglial reactivity as a candidate mechanism to be tested, not as an established response to school exclusion.

Importantly, cytokines should not be framed as uniformly harmful or exclusively pathological. Physiological IL-1 signaling contributes to hippocampal-dependent memory and synaptic plasticity, glia-derived TNF-α participates in homeostatic synaptic scaling, and endogenous IL-6 expression changes during hippocampal long-term potentiation (Avital et al., 2003; Stellwagen and Malenka, 2006; Balschun et al., 2004). The effects of IL-1β, TNF-α, IL-6, and other cytokines depend on concentration, timing, duration, cellular source, receptor distribution, developmental period, and brain region. Physiological or transient signaling may support homeostasis and plasticity, whereas excessive, prolonged, or poorly regulated signaling may interfere with synaptic and circuit function. The mechanistic concern in the proposed model is therefore persistent or dysregulated signaling rather than the mere presence of a cytokine.

## 6 Adolescent neural plasticity as a candidate vulnerability context

### 6.1 Developmental heterogeneity and non-linear brain maturation

Adolescent neurodevelopment is characterized by prolonged, heterogeneous, and measure-specific changes in brain structure and function rather than by a single uniform maturational trajectory. Longitudinal connectome research identifies marked expansion and differentiation of structural organization during adolescence, especially in transmodal association regions, while diffusion-MRI studies show that global and regional white-matter network properties can follow different developmental trajectories (Park et al., 2021; Feng et al., 2023). Together, these findings support a phased account of maturation in which developmental timing varies across systems and levels of analysis.

This heterogeneity extends to the organization of structural networks. Increasing within-module connectivity and differentiation of higher-order association networks may coexist with approximately linear change in some global properties and more variable change at regional or connection levels (Park et al., 2021; Feng et al., 2023). The adolescent brain is therefore better understood as a set of partially coordinated developmental processes than as a uniformly high-plasticity organ.

Neurochemical processes associated with excitation–inhibition balance likewise show regionally variable developmental patterns. In a 7-T magnetic resonance spectroscopic imaging study of 144 individuals aged 10–30 years, frontal glutamate levels generally decreased with age, whereas developmental changes in GABA were heterogeneous across frontal regions; the relationship between glutamate and GABA also changed from adolescence into adulthood (Perica et al., 2022). These findings provide *in vivo* evidence of continued maturation without implying a uniform excitation–inhibition ratio across the adolescent brain.

Furthermore, chronological age and pubertal development may contribute differently to neural and cognitive maturation. In a longitudinal study involving 105 participants aged 8–19 years and 227 total assessments, chronological age was associated with activation in several cognitive-control regions, whereas pubertal stage was uniquely associated with response latency and right ventrolateral prefrontal activity after age was taken into account (Ravindranath et al., 2022). Adolescence is therefore best conceptualized as a developmentally heterogeneous interval in which the timing, direction, and magnitude of change vary across neural systems, biological measures, and pubertal stages.

At a broader lifespan scale, diffusion-imaging research supports a phased rather than uniformly linear account of structural-network maturation. Data from nine datasets comprising 4,216 individuals aged from birth to 90 years identified major turning points in structural-connectome topology at approximately 9, 32, 66, and 83 years, with adolescence and young adulthood forming part of a broader epoch extending approximately from age 9 to age 32 (Mousley et al., 2025). This finding reinforces the value of developmental timing while leaving room for measure-specific trajectories.

### 6.2 Synaptic plasticity: LTP, LTD, and receptor regulation

Within this developmental context, synaptic plasticity represents a biologically plausible candidate mechanism. Experimental findings indicate that cytokine effects on synaptic function are context-dependent rather than uniformly detrimental. In mouse hippocampal slices, endogenous IL-1 signaling appeared to contribute to long-term potentiation under physiological conditions, whereas higher exogenous IL-1 concentrations inhibited LTP, demonstrating a dose- and context-dependent effect (Ross et al., 2003). The relevant mechanistic question is therefore how intensity, timing, and developmental context shape cytokine effects on plasticity.

More specifically, experimental evidence from rodent hippocampal preparations shows that elevated cytokine signaling can inhibit LTP. Recombinant IL-6 applied to rat hippocampal slices inhibited the induction of LTP in the Schaffer collateral–CA1 pathway (Li et al., 1997), while TNF-α and IL-1β suppressed chemically induced LTP in mouse hippocampal synaptosomes and neuronal cultures under the tested conditions (Prieto et al., 2019). These findings identify cytokine-sensitive steps in hippocampal potentiation that can guide future adolescent studies.

At the same time, immune signaling can contribute to forms of synaptic depression and homeostatic plasticity. In mouse hippocampal slices, TNF-receptor deletion prevented the induction of CA1 long-term depression, while interference with NF-κB signaling prevented LTD and reduced the magnitude of LTP (Albensi and Mattson, 2000). This result indicates that TNF-related signaling can participate in both forms of plasticity and should not be reduced to a simple damage model.

In addition, TNF-α regulates homeostatic synaptic scaling and neurotransmitter-receptor trafficking. Glia-derived TNF-α was required for upward synaptic scaling after prolonged activity blockade, while acute TNF-α exposure altered inhibitory synaptic strength, cell-surface GABA-A receptors, and AMPA-receptor expression through regulated receptor-trafficking mechanisms (Stellwagen and Malenka, 2006; Pribiag and Stellwagen, 2013). These studies show how immune signals can modify synaptic receptor availability and network stability.

Taken together, LTP, LTD, and receptor-trafficking findings identify measurable synaptic candidates for future developmental research. The current evidence is derived mainly from rodent brain-slice, neuronal-culture, synaptosome, and other preclinical preparations, so the key next step is to test whether comparable plasticity signatures covary with well-defined social-exclusion exposures in adolescents. This framing preserves the mechanistic value of the preclinical literature while keeping the human inference appropriately bounded.

### 6.3 Myelination and white-matter development

Structurally, white-matter microstructure and organization continue to change throughout adolescence and, for some association pathways, into early adulthood. A longitudinal diffusion-tensor imaging study followed 128 participants aged 8–28 years and identified a hierarchical pattern in which some frontocortical and cerebellar pathways approached adult-like levels during adolescence while corticolimbic association pathways matured more gradually (Simmonds et al., 2014). Earlier maturation of some pathways was associated with better cognitive-control performance, supporting a relationship between developmental timing and distributed network efficiency. These diffusion measures are sensitive to white-matter microstructure, including processes related to myelination and axonal organization, but are not direct or specific measures of myelin (Simmonds et al., 2014).

Evidence linking peripheral inflammation to white-matter microstructure comes mainly from adult clinical populations. In adults with major depressive disorder, higher serum IL-1β was associated with lower diffusion-derived white-matter measures in selected pathways (Sugimoto et al., 2018), and higher CRP was associated with reduced corticolimbic white-matter integrity (Thomas et al., 2022), while newborn-mouse experiments indicate that systemic IL-1β can disrupt oligodendrocyte maturation and myelination (Favrais et al., 2011). These findings provide convergent, but developmentally indirect, support for the possibility that sustained inflammatory signaling can influence white-matter processes. They also reinforce the value of measuring white-matter development longitudinally rather than inferring demyelination from a peripheral marker.

Overall, the evidence supports a testable hypothesis that sustained or dysregulated inflammatory signaling could influence oligodendrocyte function, myelin-related processes, or white-matter development. The adolescent-specific question is whether developmental timing and social context moderate this relationship; current studies provide the structural and cellular rationale for that test without establishing a school-exclusion effect.

### 6.4 Hippocampal plasticity and neurogenesis

Building on the synaptic mechanisms described above, hippocampal plasticity provides a regionally specific candidate context. Its interpretation should also be situated within the non-linear timing of adolescent neurodevelopment: longitudinal diffusion imaging shows that some white-matter tracts mature by late adolescence while association pathways continue to change beyond it (Lebel and Beaulieu, 2011). This developmental pattern supports treating adolescent brain systems as temporally heterogeneous rather than assuming a single maturational endpoint. Claims concerning hippocampal neurogenesis require careful interpretation because most evidence linking peripheral inflammation to this process comes from adult animal disease models rather than socially excluded adolescents. In adult male mice, experimental arthritis was accompanied by a chronic-phase reduction in doublecortin-positive dentate-gyrus cells and increased CD68-positive macrophage or microglial density, although hippocampal cytokine concentrations were not significantly altered (Rusznák et al., 2022). This study identifies a possible inflammatory correlate of adult hippocampal neurogenesis markers in mice.

Similarly, experimental colitis findings indicate that inflammatory effects on hippocampal neurogenesis are not uniformly suppressive. Acute colitis increased indices of neurogenesis but altered progenitor cell-cycle kinetics, whereas chronic colitis preserved broad neurogenesis levels while affecting migration and functional integration of newly generated dentate-gyrus neurons (Gampierakis et al., 2021). The direction of the effect therefore depends on inflammatory phase and the neurogenic outcome being measured.

Translation to human adolescence is additionally shaped by continuing disagreement about the extent and developmental trajectory of human hippocampal neurogenesis. Postmortem studies have reported both a marked childhood decline in immature neuronal markers and the persistence of comparable populations across examined adult ages (Sorrells et al., 2018; Boldrini et al., 2018). This divergence makes neurogenesis a productive research question while cautioning against treating any single postmortem estimate as a direct *in vivo* measure of adolescent function.

More recent postmortem molecular and cellular evidence supports the presence of proliferating progenitors and immature neuronal populations in the adult human hippocampus, while indicating variation with aging and Alzheimer’s disease (Disouky et al., 2026). These data add molecular detail to the human literature but do not provide a validated *in vivo* measure of neurogenesis rate or functional contribution in living adolescents.

Thus, hippocampal neurogenesis is retained as a preclinical and translational candidate mechanism within the broader plasticity framework. The most useful next studies would align well-defined educational exclusion measures with repeated inflammatory, hippocampal, cognitive, and academic assessments, allowing developmental specificity and resilience to be evaluated together.

### 6.5 Large-scale network hypotheses and reward-related processing

At the large-scale network level, repeated social-evaluative threat and dysregulated immune signaling may influence coordination among systems involved in salience and threat detection, affective distress, cognitive control, reward processing, and socially guided behavior. These systems should be treated as interacting networks rather than isolated modules, and activity in a particular region should not automatically be assigned to a single psychological process.

Adolescent social-exclusion studies provide the behavioral and neural anchor for this network hypothesis. In an fMRI study of 23 adolescents completing a Cyberball task, exclusion-related distress was associated with insula and subgenual anterior cingulate activity, whereas right ventrolateral prefrontal and ventral-striatal activity was associated with lower distress (Masten et al., 2009). Adolescents with chronic peer-rejection histories also showed heightened dorsal anterior cingulate and anterior prefrontal responses during experimentally induced or incidental exclusion (Will et al., 2016b). Together, these findings indicate that adolescent exclusion engages interacting distress, regulation, and salience systems.

Behavioral decisions after exclusion further illustrate this distributed organization. In related adolescent research, punishment of previous excluders engaged ventral-striatal, dorsolateral-prefrontal, and parietal regions, while adolescents with chronic rejection histories showed greater dorsal-striatal and lateral-prefrontal activity when they refrained from punishment and allocated resources equally (Will et al., 2016a). These results extend the framework from distress to action selection and social decision-making.

Adult inflammatory-challenge studies provide a complementary mechanistic reference. Endotoxin altered neural responses to negative and positive social feedback in amygdala, cingulate, prefrontal, and ventral-striatal regions, while other acute-challenge studies reported changes in resting-state connectivity (Muscatell et al., 2016; Labrenz et al., 2016). Their value is to identify experimentally manipulable network targets for developmental research, not to substitute adult immune challenges for adolescent school-based evidence.

The inflammation–reward hypothesis can therefore be stated as a concise causal chain: an immune signal may alter striatal and prefrontal reward processing; the altered balance between reward anticipation, feedback, and social evaluation may bias motivation or action selection; and those behavioral changes may shape engagement with peers and school tasks. Adult endotoxin studies show that the direction of this effect depends on reward type, processing phase, and participant sex, while adolescent studies link stress-related IL-6 or CRP to context-dependent reward behavior and nucleus-accumbens responses (Moieni et al., 2019; Kuhlman et al., 2023; Yuan et al., 2024). This chain is biologically coherent and testable, but it remains an indirect hypothesis for school-based exclusion during adolescence.

Taken together, adolescent exclusion studies and adult inflammation experiments implicate partially overlapping systems, including the anterior cingulate cortex, insula, amygdala, ventral striatum, and prefrontal cortex. This overlap is theoretically informative because it links social distress, reward-related behavior, and cognitive control within one network-level framework. The studies nevertheless examine different ages, exposures, immune manipulations, tasks, and outcomes. Figure 2 is therefore presented after the outcome evidence in Section 7 as a synthesis of the inferential framework rather than as a premise for causal interpretation.

**Figure 2:**
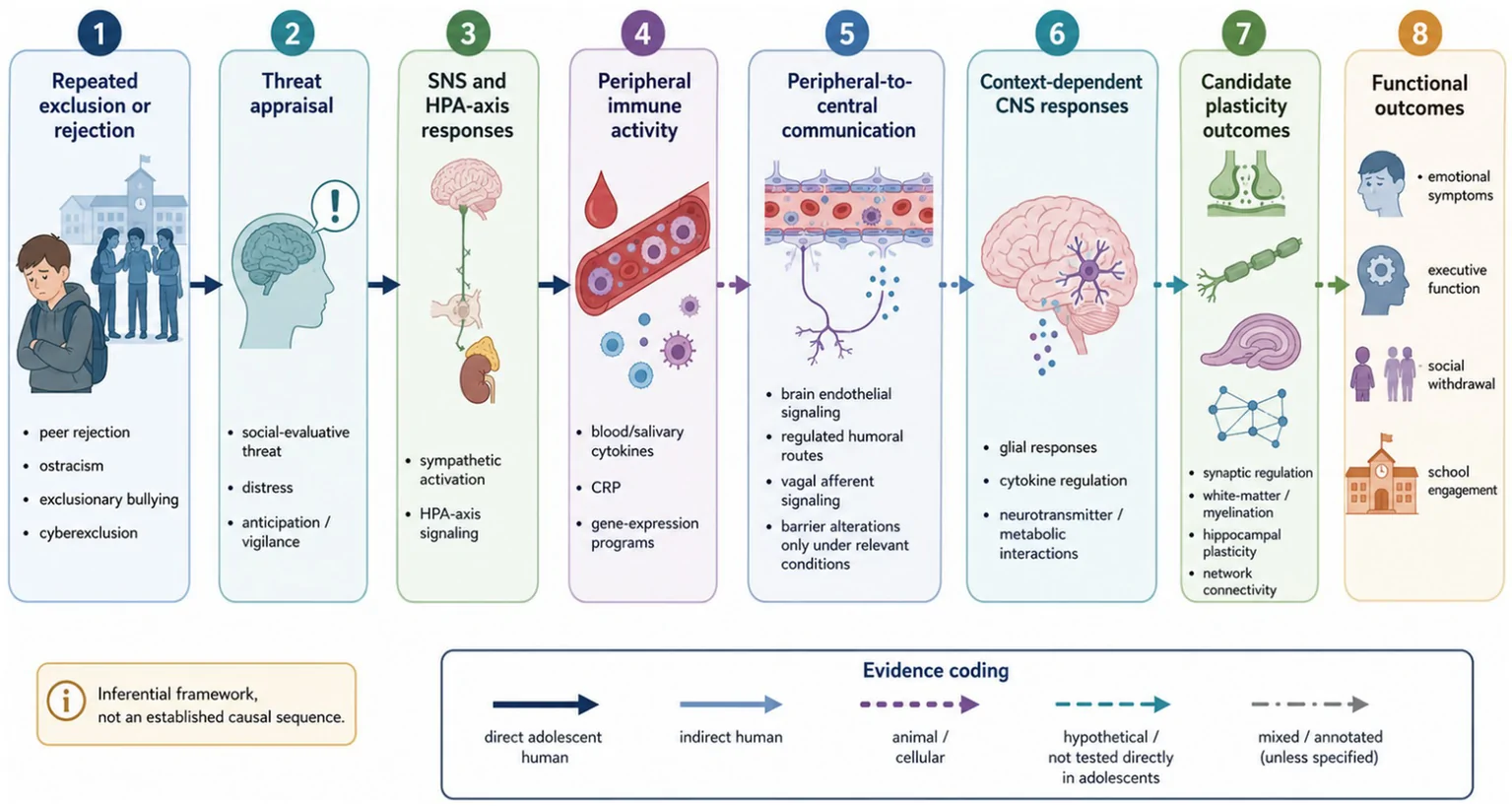
Candidate pathway linking repeated social-evaluative threat with peripheral immune activity, central neuroimmune signaling, adolescent neural plasticity, and functional outcomes.

## 7 Psychological, cognitive, and educational outcomes

### 7.1 Neurotransmitter, metabolic, and reward-processing correlates

At the intermediary level, neurotransmitter and metabolic pathways are treated as interacting correlates rather than as direct and sufficient explanations of depression, anxiety, or other psychological outcomes. Available evidence concerns serotonergic transport, dopamine-precursor metabolism and presynaptic dopamine function, glutamate-related metabolism, and the kynurenine pathway. These mechanisms have mainly been examined in adult clinical samples, experimental immune-challenge studies, and animal models, while adolescent work has begun to connect peripheral inflammation with reward-related behavior and neural activity.

Human evidence suggests a possible relationship between peripheral inflammatory activity and serotonergic function. Circulating TNF-α was positively associated with brainstem serotonin-transporter availability in healthy adult women and in patients with psoriasis or psoriatic arthritis; etanercept treatment in the clinical group was subsequently associated with reduced transporter availability (Krishnadas et al., 2016). This study identifies an inflammation–serotonin association in adults without establishing a uniform change in serotonin availability after adolescent social stress.

Dopamine-related processes provide a complementary example. In adults receiving interferon-α for hepatitis C, an increased plasma phenylalanine-to-tyrosine ratio was associated with lower cerebrospinal-fluid dopamine and homovanillic-acid concentrations, and reduced cerebrospinal-fluid tetrahydrobiopterin was associated with higher IL-6 (Felger et al., 2013). These findings support a biologically plausible route from cytokine signaling to dopamine-precursor metabolism, while the clinical treatment context limits direct translation to healthy adolescents.

Reward-related findings further connect inflammatory signaling with behavior. Interferon-α treatment in adults with hepatitis C was associated with reduced bilateral ventral-striatal responses during reward processing, altered fluorodopa uptake and turnover in the caudate and putamen, and behavioral symptoms including anhedonia, depression, and fatigue (Capuron et al., 2012). The findings support an inflammation–dopamine–reward association in an adult clinical immune-activation model.

The kynurenine pathway and glutamate–glutamine metabolism provide additional candidate links. In an endotoxin experiment, kynurenine and quinolinic acid were associated with depressed mood within the endotoxin group, but the findings did not establish mediation after correction for multiple comparisons; interferon-α treatment also altered glutamine-related metabolism in the pregenual anterior cingulate cortex (Kruse et al., 2019; Taylor et al., 2014). These results identify metabolic processes that can be measured alongside reward and affective outcomes.

Adolescent-specific evidence is limited but informative. Early-life adversity moderated the association between stress-related plasma IL-6 increases and reward or risk processing (Kuhlman et al., 2023), whereas higher CRP was associated with reduced nucleus-accumbens activation during reward outcomes among adolescents with greater early-life stress (Yuan et al., 2024). These studies support context-dependent inflammation–reward associations in adolescence and motivate direct tests using school-exclusion exposures.

### 7.2 Executive function and academic participation

Executive functions comprise partially separable cognitive processes—including working memory, inhibitory control, cognitive flexibility, planning, and performance monitoring—that support goal-directed behavior in educational settings. In 151 male adolescents aged 11–16 years, executive-function components predicted reading, mathematics, science, and social-studies achievement after general intellectual functioning was taken into account (Latzman et al., 2010). The findings support the educational relevance of executive processes while leaving room for domain- and context-specific effects.

Acute exclusion can influence cognitive performance during adolescence, although the direction and magnitude vary by age and cognitive domain. Following a virtual ball-tossing exclusion task, younger adolescent girls showed reduced verbal-working-memory accuracy, whereas middle adolescents and adults did not show the same effect and visuospatial working memory was unaffected (Fuhrmann et al., 2019). This pattern supports a short-term, age- and domain-specific effect rather than generalized executive dysfunction.

Peer ecology also shapes academic participation. In 5,991 students followed from sixth to eighth grade across 26 middle schools, peer rejection was associated with later academic difficulties, but the association was weaker in schools where popular peers displayed stronger academic engagement (Lessard and Juvonen, 2022). The finding identifies school-level norms as a potentially modifiable context that can buffer educational risk.

Peripheral inflammatory activity may represent an additional correlate of executive functioning and academic performance. In a population-based sample of 1,066 Dutch adolescents, higher blood CRP was associated with poorer future executive functioning (Mac Giollabhui et al., 2021); in a separate cross-sectional sample of 244 adolescents, higher TNF-α and CRP were associated with selected academic and verbal-ability measures (Adelantado-Renau et al., 2020). These results support including inflammatory measures in carefully designed developmental studies while retaining attention to multiple pathways and baseline differences.

Taken together, executive and academic outcomes are multiply determined. Social exclusion may influence academic functioning through acute cognitive distraction, threat monitoring, reduced motivation, lower classroom participation, peer-group norms, emotional symptoms, absenteeism, and other behavioral pathways. Peripheral inflammatory activity may be one correlate or vulnerability marker within this network, while the strongest practical implication is that school climate and participation are modifiable targets in their own right.

### 7.3 Emotion regulation, rejection sensitivity, and social withdrawal

Emotion regulation and social-information processing provide a better-supported psychological bridge between social exclusion and subsequent adjustment than a direct inflammation-to-behavior explanation. Rejection sensitivity refers to the tendency to anxiously or angrily expect, readily perceive, and overreact to interpersonal rejection. It can influence how adolescents interpret ambiguous peer behavior, attribute responsibility for rejection, and select withdrawal, avoidance, or retaliatory responses.

Longitudinal evidence shows that rejection-related responses are heterogeneous. In a three-wave study of 713 young adolescents, self-blame was associated with withdrawal, withdrawal and anxious rejection sensitivity predicted later depressive symptoms, and angry rejection sensitivity, peer-blame, and retaliation were associated with later aggressive behavior (Zimmer-Gembeck et al., 2016). These patterns distinguish internalizing and externalizing routes rather than treating rejection sensitivity as a single process.

Emotion regulation can modify these associations. In 590 Chinese children and early adolescents, rejection sensitivity predicted later internalizing difficulties, while better emotion regulation buffered prospective links with peer and emotional problems (Ding et al., 2021). Emotion regulation is therefore both a potential moderator and a practical intervention target.

Neuroimaging evidence provides a complementary bridge between subjective experience and brain function. In 23 adolescents completing Cyberball, insula and subgenual anterior cingulate activity tracked exclusion-related distress, while right ventrolateral prefrontal and ventral-striatal activity was associated with lower distress; greater rejection sensitivity was associated with greater neural evidence of distress (Masten et al., 2009). These findings connect appraisal, regulation, and regional neural responses without requiring a single biological mechanism.

Finally, victimization and social withdrawal appear interrelated but not governed by a universal escalating cycle. Multi-wave studies found concurrent associations and between-person differences, but did not consistently identify robust cross-lagged effects in which victimization caused later rejection sensitivity or vice versa; population-based work similarly supports separating within-person from between-person processes (Kellij et al., 2024; Barzeva et al., 2020). This distinction helps identify when withdrawal may reduce immediate threat and when it may reduce access to support.

Taken together, the evidence supports a conditional psychological pathway in which rejection expectations, self- or peer-directed attributions, emotion-regulation capacity, and coping responses shape adolescents’ reactions to exclusion. These processes identify concrete targets for prevention and support: reducing hostile attribution, strengthening regulation, and preserving access to supportive relationships. Inflammatory mediation remains a testable biological question rather than a necessary assumption of the psychological pathway.

### 7.4 Long-term outcomes: risk rather than inevitability

Beyond immediate responses, long-term psychological, social, and educational outcomes are best described as probabilistic risks rather than inevitable consequences. Prospective cohort studies indicate that peer victimization during adolescence is associated with later depression and anxiety, but the magnitude and meaning of these associations vary across exposure frequency, reporter, developmental period, outcome definition, and statistical adjustment. The sequential evidence below therefore moves from prospective risk, to heterogeneity and resilience, to possible biological moderators and broader neuropsychiatric claims.

Prospective cohort evidence illustrates this risk pattern. In the Avon Longitudinal Study of Parents and Children, frequent peer victimization at age 13 was associated with higher odds of depression at age 18, with depression identified in 14.8% of frequently victimized participants compared with 5.5% of those reporting no victimization (Bowes et al., 2015). In the same broader cohort, frequently victimized adolescents were approximately two-and-a-half times more likely to meet criteria for an anxiety disorder at age 18 after adjustment for prior emotional symptoms and family-related confounders (Stapinski et al., 2014). Together, these studies support prospective risk associations while leaving causal pathways open to developmental, familial, and contextual explanation.

At the same time, long-term associations are not uniform across studies or reporters. In the Tracking Adolescents’ Individual Lives Survey, parent-reported peer victimization showed relatively stable associations with later internalizing symptoms, thought problems, and somatic complaints, whereas self-reported and peer-reported victimization did not consistently predict adult maladjustment; teacher-reported associations were weaker and largely attenuated after parent reports were considered (Kretschmer et al., 2025). This evidence makes measurement perspective part of the substantive interpretation rather than a technical afterthought.

Moreover, elevated risk does not imply inevitability. Many exposed adolescents do not develop persistent psychiatric symptoms, and outcomes may be modified by exposure duration, severity, prior mental health, family adversity, friendship and parental support, school climate, coping, pubertal development, and access to intervention. Formulations such as “increased vulnerability” and “higher prospective risk” therefore better reflect the literature than claims of permanent psychiatric damage.

Inflammatory reactivity may identify a subgroup with greater vulnerability to interpersonal stress. In a nine-month prospective study of 116 girls at elevated risk for psychopathology, greater interpersonal life stress predicted increases in depressive symptoms only among participants with stronger salivary TNF-α or IL-1β responses to a laboratory social stressor; the interaction was not significant for IL-6 (Slavich et al., 2020). The result supports moderation as a useful model for individual differences in risk and highlights the need to measure appraisal, coping, and developmental context alongside biomarkers.

Accordingly, persistent inflammatory reactivity can be considered one possible vulnerability marker or interacting process, alongside threat appraisal, rejection sensitivity, emotion regulation, sleep disturbance, reduced activity, social withdrawal, academic disengagement, prior psychopathology, and family or socioeconomic adversity. This multilevel interpretation keeps the biological signal connected to the social and behavioral processes through which long-term outcomes emerge.

Attention and behavioral symptoms require the same developmental caution. Pre-existing inattention, impulsivity, social difficulties, or other neurodevelopmental characteristics may increase the likelihood of peer rejection or victimization. Conversely, peer victimization may co-occur with or prospectively relate to attention and behavioral symptoms in some cohorts. These bidirectional possibilities argue against claims that social exclusion causes ADHD or a broad range of neuropsychiatric disorders without direct measurement of exposure, timing, diagnosis, and mechanism.

Finally, epigenetic evidence remains hypothesis-generating and preclinical. In adult mice exposed to chronic social-defeat stress, selected hippocampal BDNF transcripts were downregulated alongside increased repressive histone methylation at corresponding BDNF promoters; chronic imipramine reversed several molecular changes (Tsankova et al., 2006). This experiment demonstrates stress-related chromatin regulation in an adult mouse model and provides a candidate molecular process for future developmental work.

More developmentally relevant animal evidence comes from a mouse study in which social stress during early adolescence was followed by adult social avoidance and cognitive inflexibility. These outcomes were accompanied by reduced total and exon-specific BDNF expression and increased H3K9 dimethylation near the BDNF IV promoter in the medial prefrontal cortex, without corresponding changes in several other histone marks or in DNA methylation at the examined promoter (Xu et al., 2018). Together, the studies identify selective, testable molecular hypotheses while showing why social-defeat exposure in mice cannot be treated as equivalent to educational social exclusion in humans.

Taken together, Sections 4–7 support an evidence-graded candidate pathway in which social-evaluative threat, immune signaling, adolescent neural plasticity, and functional outcomes are connected through interacting rather than inevitable processes. Figure 2 summarizes this framework after the outcome evidence so that the model is read as a synthesis of the review rather than as a causal premise.

Figure 2 integrates the reviewed levels of evidence. The chronological sequence runs from repeated exclusion or rejection, through threat appraisal and autonomic or endocrine responses, peripheral immune activity, brain endothelial, humoral, and vagal signaling, context-dependent glial and cytokine responses, candidate effects on synaptic regulation, myelination, hippocampal plasticity, and network function, and finally psychological, cognitive, and educational outcomes. Arrow styles are defined in the evidence-coding legend: solid dark arrows indicate direct adolescent human evidence; solid light arrows indicate indirect human evidence; dotted arrows indicate animal or cellular evidence; dashed arrows indicate hypothetical links not tested directly in adolescents; and mixed or annotated arrows indicate heterogeneous evidence or links for which a single category is not specified. The figure represents an inferential framework rather than an established causal sequence. Source: Created by the authors using original schematic artwork; no third-party copyrighted material is reproduced.

## 8 Evidence-based school implications

Educational recommendations are organized according to the mechanism directly targeted and the strength of the supporting evidence. Interventions that prevent exclusion or modify the peer ecology are most closely matched to the proposed exposure, whereas psychological-support and health-promoting interventions operate through broader pathways. This distinction preserves the practical value of school-based action while keeping biological mechanisms as testable secondary hypotheses.

### 8.1 Primary prevention: reducing exclusion

The first priority is to prevent and reduce exclusion, peer rejection, and exclusionary forms of bullying. An updated systematic review and meta-analysis concluded that school-based anti-bullying programs produce statistically significant reductions in both bullying perpetration and victimization, although effect sizes vary substantially across programs, settings, implementation conditions, and study designs (Gaffney et al., 2021). These findings support school-wide prevention, but they do not imply that every program component is independently effective or that generic anti-bullying procedures adequately address ostracism, relational exclusion, and cyberexclusion.

Programs should therefore define the targeted behavior explicitly. Bullying is characterized by intentionality, repetition, and a power imbalance, whereas relational aggression can include exclusion, friendship manipulation, rumor spreading, and other behaviors intended to damage social relationships (Menesini and Salmivalli, 2017). School monitoring systems should consequently record relational and digital exclusion separately from physical or verbal aggression, because an intervention may reduce overt bullying while leaving subtle exclusionary processes undetected.

Cooperative learning provides one classroom-level strategy for modifying peer relations rather than responding only after victimization has occurred. In a cluster-randomized middle-school study, structured cooperative learning improved positive peer relations and reduced victimization, bullying, emotional problems, and academic disengagement relative to usual instructional practice (Van Ryzin and Roseth, 2018). A subsequent randomized study reported that cooperative learning was associated with improved peer relations and empathy and with lower bullying, supporting the possibility that classroom structure can alter the social processes sustaining exclusionary behavior (Van Ryzin and Roseth, 2019). These findings are relevant to peer ecology, but they should not be generalized to all forms of school exclusion without replication across cultures, age groups, and implementation models.

Practical primary-prevention systems may include repeated assessment of peer relations, confidential reporting routes, procedures for responding to relational and cyber exclusion, teacher training in recognizing students who are persistently omitted from group activity, structured opportunities for cooperative interaction, and follow-up after an incident. These elements should be implemented as an integrated safeguarding and inclusion system rather than presented as individually proven components. Schools should document intervention fidelity, student uptake, staff response time, and unintended effects, including whether reporting procedures increase visibility without improving protection.

These strategies act primarily on the exposure and social environment. Their primary value is the reduction of exclusion and improvement of students’ safety and participation; whether they also alter inflammatory physiology is an empirical question that should be tested in appropriately designed studies.

### 8.2 Secondary prevention: strengthening belonging and coping

The second priority is to strengthen psychological and social buffers among students who are exposed to rejection, anticipate exclusion, or show early emotional or educational difficulties. Universal school-based social and emotional learning programs have been associated with improvements in social–emotional skills, attitudes, behavior, and academic performance (Durlak et al., 2011). Follow-up meta-analysis further indicated that benefits from school-based social and emotional learning interventions can persist after program completion, although effects vary across outcomes and implementation quality (Taylor et al., 2017). These programs may strengthen emotion regulation and coping, but they should not be assumed to reduce inflammation or prevent neural changes unless those outcomes are directly measured.

Supportive teacher-student relationships are also relevant because relational quality is associated with student engagement and achievement. A meta-analytic update of 189 studies involving 249,198 students found that positive and negative affective teacher-student relationships were related to engagement and achievement, with engagement functioning as an important explanatory process (Roorda et al., 2017). This literature supports teacher responsiveness and relational support as educational targets, but it is largely correlational and should not be interpreted as evidence that teacher support biologically reverses the effects of exclusion.

Mentoring can provide an additional relationship-based support for students who lack reliable peer or adult connections. A meta-analysis of intergenerational, one-to-one youth mentoring programs found modest overall benefits across youth outcomes, with effectiveness depending on program practices and participant characteristics (Raposa et al., 2019). Mentoring should therefore be presented as a potentially useful support rather than a uniformly effective remedy, and programs should monitor relationship quality, duration, premature termination, and access for students who are most socially marginalized.

Accessible school mental-health services may be required when exclusion is accompanied by clinically meaningful anxiety, depression, self-harm risk, or functional impairment. A meta-analysis of rigorous randomized trials found that school-based interventions targeting depression or anxiety can improve symptoms, with effects varying by intervention type, delivery personnel, school level, and prevention or treatment focus (Zhang et al., 2023). A broader meta-analysis of brief school-based psychosocial interventions similarly reported small but statistically significant improvements in mental health or wellbeing at follow-up, while emphasizing the need to optimize real-world implementation (Cohen et al., 2024). Services should be integrated with referral and safeguarding systems rather than used as a substitute for changing an exclusionary school environment.

School belonging and connectedness should be measured as outcomes rather than presumed to improve. A recent scoping review identified 24 adolescent school-connectedness intervention studies across 12 countries; strategies were heterogeneous, and positive effects were reported in only a subset of interventions (Attri et al., 2025). This evidence supports targeted evaluation of belonging, student engagement, and implementation fidelity rather than assuming that any classroom or mental-health program will increase connectedness.

The proposed relevance of social–emotional learning, mentoring, teacher support, and mental-health services to inflammatory physiology is a testable secondary question. Their immediate educational value lies in strengthening coping, relationships, belonging, and access to care, and these outcomes should be evaluated directly.

### 8.3 Supportive health-promoting practices

The third intervention level concerns general physiological and psychological regulation. Regular physical activity has established health benefits and may influence some peripheral inflammatory markers. A systematic review and meta-analysis of 38 exercise studies involving 2,043 children and adolescents reported reductions in IL-6 and C-reactive protein, but not TNF-α; subgroup findings suggested that effects differed by age and exercise modality (Khalafi et al., 2024). These results justify presenting physical activity as a general health-promoting practice, but they do not show that exercise reverses inflammatory or neural effects caused specifically by educational social exclusion.

Adequate sleep should also be promoted because sleep supports mental health, learning, and physiological regulation. However, a systematic review of observational studies in children and adolescents concluded that the available literature did not provide clear evidence of a consistent association between sleep duration or quality and inflammatory biomarkers (Medeiros-Oliveira et al., 2023). Sleep promotion should therefore be recommended on broad developmental and mental-health grounds, not described as a proven anti-inflammatory treatment for excluded adolescents.

Balanced nutrition may contribute to general metabolic and immune health, but evidence in young people remains heterogeneous. A systematic review found that healthier dietary patterns, fruit, vegetables, whole grains, and unsaturated fats were often associated with more favorable inflammatory profiles, whereas Western dietary patterns and higher saturated-fat intake were often associated with less favorable profiles; the authors also identified substantial variation in design, dietary assessment, biomarkers, and adjustment for adiposity and health status (Bujtor et al., 2021). Nutritional guidance should consequently focus on overall dietary quality and health rather than claim that a specific diet blocks the neuroimmune consequences of school exclusion.

Mindfulness and stress-management programs require particularly cautious presentation. A meta-analysis of school-based stress-reduction programs found an overall reduction in adolescent psychological stress, but statistically significant effects were concentrated in selected rather than universal student samples (van Loon et al., 2020). In the large MYRIAD cluster-randomized trial, universal school-based mindfulness training was not superior to usual school provision for preventing mental-health problems or promoting wellbeing, indicating that acceptability, engagement, developmental fit, and implementation context matter (Kuyken et al., 2022). Mindfulness should therefore be offered as one potentially useful option, not mandated as a universal anti-inflammatory intervention.

Collectively, physical activity, sleep support, nutrition, mindfulness, and stress-management practices may improve general wellbeing or peripheral physiology under some conditions. They are best presented as complementary supports whose effects on any exclusion-related neuroplasticity process remain to be tested.

### 8.4 Measurement and evaluation

School intervention studies should use a prespecified multilevel measurement plan that distinguishes exposure, mechanism, process, and outcome variables. Exposure measures may include the frequency and duration of ostracism, sociometric peer rejection, exclusionary or relational bullying, cyberexclusion, and objective indicators of social isolation. Because these constructs are not interchangeable, each measure should correspond to the intervention target and should identify the reporter, time frame, and setting.

Process and contextual outcomes should include perceived school belonging or connectedness, social support, teacher-student relationship quality, friendship formation, help-seeking, perceived safety, implementation fidelity, attendance at intervention sessions, and student acceptability. The literature on adolescent school-connectedness interventions shows wide variation in definitions, measures, intervention strategies, and implementation, reinforcing the need to specify the construct and instrument rather than use belonging as an undefined outcome (Attri et al., 2025).

Psychological outcomes may include exclusion-related distress, loneliness, rejection sensitivity, emotion regulation, depressive symptoms, anxiety symptoms, and perceived stress. Educational outcomes may include attendance, lateness, disciplinary events, classroom participation, school engagement, executive-function tasks, grades, standardized attainment, and school continuation. Baseline performance and prior mental-health symptoms should be measured so that post-intervention differences are not interpreted without accounting for pre-existing variation.

Studies should also account for the clustered structure of school data. Students are nested within classrooms and schools, and peer processes can spread across social networks. Cluster randomization or multilevel analysis is generally more appropriate than treating all students as statistically independent. Intervention fidelity, contamination between conditions, staff turnover, differential attrition, and variation in school climate should be reported because they can alter both effectiveness estimates and interpretation.

Biological measures such as CRP, IL-6, TNF-α, or salivary cytokine reactivity should be included only when they answer a prespecified mechanistic question and the study is adequately powered for biomarker analyses. Salivary inflammatory markers require standardized collection, storage, processing, assay, and reporting procedures, with attention to oral health, visible blood contamination, collection time, flow rate, assay detection limits, and correspondence with blood-based markers (Szabo et al., 2021). Best-practice recommendations further emphasize that salivary cytokines should not be interpreted as direct substitutes for systemic or central inflammation and that investigators should document oral and general health, collection conditions, assay performance, and relevant covariates (Riis et al., 2021).

At minimum, biomarker protocols should record acute infection or illness, recent vaccination, chronic inflammatory or metabolic conditions, oral disease, body mass index or adiposity, pubertal stage, biological sex, menstrual factors when relevant, sleep, physical activity, tobacco or substance exposure, food intake around sampling, prescription and non-prescription medication, and the time of day. These variables should not be entered automatically as interchangeable covariates; their role should be justified using a prespecified causal model.

Repeated biological sampling is preferable to a single post-intervention value when the objective is to test temporal mediation. A credible mediation design would require evidence that the intervention first reduces exclusion or threat appraisal, that subsequent change occurs in the proposed biological mediator, and that later change occurs in psychological, cognitive, or educational outcomes. Concurrent correlations between a cytokine and an outcome do not establish mediation, and peripheral inflammatory change does not demonstrate central neuroimmune or neural-plasticity change.

Finally, intervention studies should report adverse events and equity-related outcomes. A program may benefit students with established friendships while failing to reach highly rejected, neurodivergent, disabled, minoritized, or socioeconomically disadvantaged students. Analyses should therefore examine differential participation and effects without treating subgroup findings as definitive when sample sizes are insufficient. The principal evaluation question should remain whether the intervention reduces exclusion and improves meaningful student functioning; biological outcomes are secondary unless the trial was explicitly designed and powered to test them. Table 3 outlines intervention targets and recommended evaluation indicators.

**Table 3:** Intervention targets and recommended evaluation indicators.

Intervention level	Examples	Primary target	Recommended evaluation indicators	Evidence-based qualification	Supporting evidence
Primary prevention: reduce exclusion	School-wide anti-bullying procedures; explicit monitoring of relational and cyber exclusion; confidential reporting; rapid response and follow-up	Exposure itself: peer rejection, ostracism, exclusionary bullying, and cyberexclusion	Peer-reported or self-reported exclusion frequency; bullying incidents; sociometric rejection; response time; recurrence; perceived safety; school belonging	These strategies are most closely matched to the proposed exposure. Effects vary by program, school context, implementation quality, and behavior targeted. They should not be described as anti-inflammatory interventions unless biological outcomes are measured and temporal mediation is tested.	Menesini and Salmivalli (2017); Gaffney et al. (2021)
Primary prevention: modify peer ecology	Structured cooperative learning; peer-support systems; inclusive group-work procedures; teacher facilitation of peer interaction	Peer norms, opportunities for positive contact, empathy, and classroom social structure	Positive peer relations; victimization and bullying; friendship formation; empathy; academic engagement; intervention fidelity	Randomized middle-school studies support cooperative learning as a promising strategy, but effects should not be generalized to every form of exclusion or every cultural setting.	Van Ryzin and Roseth (2018); Van Ryzin and Roseth (2019)
Secondary prevention: strengthen coping and belonging	Social–emotional learning; emotion-regulation training; school-belonging initiatives; supportive teacher-student relationships	Threat appraisal, coping, emotional regulation, social support, and connectedness	Emotion regulation; rejection sensitivity; distress; loneliness; school belonging; help-seeking; classroom engagement; depressive and anxiety symptoms	SEL and relational-support interventions can improve social–emotional and educational outcomes, but their relevance to inflammatory physiology is indirect and should be tested rather than assumed.	Durlak et al. (2011); Taylor et al. (2017); Roorda et al. (2017); Attri et al. (2025)
Secondary prevention: targeted relational and mental-health support	Mentoring; accessible school mental-health services; indicated anxiety or depression interventions; safeguarding and referral pathways	Reliable supportive relationships, clinically meaningful symptoms, and functional impairment	Mentoring relationship quality and duration; service uptake; symptom change; self-harm risk; attendance; participation; referral completion; adverse events	Average effects are generally modest and depend on program quality, implementation, and student need. Psychological services must not substitute for changing an exclusionary school environment.	Raposa et al. (2019); Zhang et al. (2023); Cohen et al. (2024)
Supportive health-promoting practices	Regular physical activity; sleep support; balanced nutrition; appropriately delivered mindfulness or stress-management programs	General psychological and physiological regulation rather than exclusion-specific mechanisms	Physical activity; sleep duration and quality; dietary quality; perceived stress; wellbeing; optional peripheral inflammatory markers	These practices may support general health and may influence inflammation in some contexts. Current evidence does not show that they reverse neural-plasticity alterations caused by school exclusion. Mindfulness should not be presented as universally effective.	Khalafi et al. (2024); Medeiros-Oliveira et al. (2023); Bujtor et al. (2021); van Loon et al. (2020); Kuyken et al. (2022)
Multilevel intervention evaluation	Cluster-randomized or longitudinal school studies with exposure, process, psychological, educational, and biological measures	Intervention effectiveness, temporal ordering, mechanisms, equity, and implementation	Exposure: exclusion, rejection, bullying, cyberexclusion. Process: belonging, support, fidelity. Psychological: distress, loneliness, emotion regulation. Educational: attendance, engagement, executive function, attainment. Biological: CRP, IL-6, TNF-α or salivary cytokine reactivity when justified.	Constructs must be measured separately. School clustering, baseline differences, attrition, contamination, pubertal stage, adiposity, infection, medication, sleep, oral health, sampling time, and assay performance should be addressed. Peripheral biomarkers do not establish central neuroinflammation.	Szabo et al. (2021); Riis et al. (2021)

Biological outcomes should be considered secondary unless the study is specifically designed and powered to test a mechanistic hypothesis. Changes in peripheral inflammatory markers do not demonstrate central neuroinflammation or altered neural plasticity.

## 9 Discussion: evidence, mechanisms, and limitations

The reviewed evidence makes a constructive contribution to a fragmented literature. Direct adolescent studies anchor the framework at the level of peripheral inflammatory reactivity and social or educational outcomes, while developmental and neuroimmune studies identify candidate processes that can be tested across levels. The value of the model lies not in claiming a completed causal chain, but in specifying which links are established, which are conditional, and which measurements are needed to connect them.

Within this framework, the model is conditional rather than deterministic. Acute inflammatory responses can be adaptive and time-limited, and cytokines have physiological roles in plasticity. Microglial reactivity can support homeostasis or adaptation depending on timing and context. The consequences of exclusion are likely to differ according to duration, intentionality, perceived controllability, prior adversity, sex, pubertal stage, social support, coping, sleep, physical health, and school climate.

At the functional level, depressive symptoms, anxiety, low self-esteem, social withdrawal, and reduced school engagement are unlikely to arise from a single cytokine or neurotransmitter alteration. They may reflect interactions among social-threat appraisal, learned expectations, HPA-axis and autonomic responses, serotonergic and dopaminergic processes, glutamate and kynurenine metabolism, sleep, behavior, and the wider social environment. Inflammation may contribute to, moderate, or accompany these processes in some adolescents, particularly those with additional vulnerabilities, but it should not be presented as a universal mediator or sufficient biological cause of emotional or educational impairment.

A careful interpretation also requires attention to alternative explanations. Depressive symptoms, anxiety, adiposity, sleep disturbance, infection, medication use, substance use, and socioeconomic adversity may increase both the probability of social exclusion and inflammatory activity. Reverse causation is also possible: pre-existing psychological or behavioral difficulties may contribute to peer rejection, while inflammatory or health conditions may reduce social participation. These possibilities make temporal ordering and repeated measurement central to future tests of the framework.

### 9.1 Interpretive boundaries and limitations

The principal interpretive boundaries are methodological rather than evidence failures. The literature uses non-equivalent exposure constructs, age ranges, reporters, biological specimens, and outcome measures; acute exclusion tasks do not fully model chronic classroom dynamics, and peripheral or salivary markers do not establish central neuroinflammation. Cross-sectional and short-term designs limit temporal inference, while adult clinical and animal models provide mechanistic leverage with limited ecological equivalence to subtle peer exclusion in schools. Because this review is narrative, it does not provide exhaustive coverage or a formal risk-of-bias assessment. These constraints do not negate the observed associations; they define the scope of inference and identify priorities for direct longitudinal tests.

## 10 Future research priorities

Future studies should test the proposed pathway prospectively in adolescent samples using repeated measures of peer exclusion, inflammatory activity, brain development, and functioning. At minimum, designs should distinguish acute exclusion from chronic peer rejection, record pubertal stage rather than age alone, and measure relevant confounders including body mass index, infection, medication, sleep, physical activity, socioeconomic adversity, and prior mental-health symptoms.

Multimodal longitudinal studies are needed to determine temporal ordering. A strong design would measure school exclusion and belonging, repeated blood or saliva inflammatory markers, neuroimaging of prefrontal–limbic and white-matter development, executive function, emotional symptoms, and academic engagement at multiple time points. Mediation should be tested only when the temporal sequence and measurement reliability are adequate.

Intervention research should first evaluate whether reducing relational exclusion and increasing school belonging improves psychological and educational outcomes. Biological measures can then be added to test whether changes in peripheral inflammatory activity accompany improvement. Randomized or cluster-randomized school trials, preregistered analyses, sufficiently powered subgroup tests, and long-term follow-up are priorities. Evidence should also be stratified by sex, gender, pubertal timing, neurodevelopmental profile, and social disadvantage.

Finally, future work should seek direct indicators of central neuroimmune function that are ethically and methodologically feasible in adolescents. Until such measures are available, peripheral inflammation, neural activity, and microglial mechanisms should be treated as related levels of evidence rather than interchangeable biological outcomes.

## 11 Conclusion

Social exclusion in educational settings is a meaningful interpersonal stressor with established associations with adolescent distress, social functioning, and school participation. A smaller body of adolescent research further suggests that peer victimization and interpersonal stress can be associated with heightened peripheral inflammatory reactivity in vulnerable youths. Developmental neuroscience and neuroimmunology provide plausible mechanisms through which sustained dysregulated immune signaling could influence neural systems undergoing adolescent reorganization. Nevertheless, direct evidence for a continuous causal pathway from school exclusion to central neuroinflammation, altered neural plasticity, and long-term academic or psychiatric outcomes is currently insufficient. The principal contribution of this review is therefore an evidence-graded framework that separates established associations from indirect mechanisms and testable hypotheses. Educational practice should prioritize preventing exclusion, strengthening belonging and social support, and providing accessible psychological support, while general health-promoting interventions should be described as complementary. Progress will depend on longitudinal, multimodal, developmentally specific studies that test temporal ordering and alternative explanations.
